# Lung Delivery of Lactose-Free Microparticles Loaded with Azithromycin for the Treatment of Bacterial Infections

**DOI:** 10.3390/pharmaceutics17060770

**Published:** 2025-06-11

**Authors:** Gracia Molina, Dolores R. Serrano, María Auxiliadora Dea-Ayuela, Carmina Rodriguez, Elena González-Burgos, Brayan J. Anaya

**Affiliations:** 1 Pharmaceutics and Food Technology Department, Faculty of Pharmacy, Universidad Complutense de Madrid, Plaza Ramón y Cajal s/n, 28040 Madrid, Spain; gramolin@ucm.es; 2 Instituto Universitario de Farmacia Industrial, Faculty of Pharmacy, Universidad Complutense de Madrid, 28040 Madrid, Spain; 3 Department of Pharmacy, Universidad Cardenal Herrera-CEU, CEU University, 46115 Valencia, Spain; mdea@uchceu.es; 4 Department of Microbiology and Parasitology, Faculty of Pharmacy, Universidad Complutense de Madrid (UCM), 28040 Madrid, Spain; carmina@farm.ucm.es; 5 Department of Pharmacology, Pharmacognosy and Botany, Faculty of Pharmacy, Universidad Complutense de Madrid (UCM), 28040 Madrid, Spain; elenagon@ucm.es

**Keywords:** azithromycin, dry powder inhaler, lactose-free, microparticles, pulmonary drug delivery

## Abstract

**Background/Objectives:** Respiratory bacterial infections remain a significant global health challenge, with effective drug delivery to the lungs being crucial for successful treatment. This study aimed to develop a lactose-free dry powder inhaler (DPI) formulation containing azithromycin (AZM) microparticles for enhanced pulmonary delivery. **Methods:** Using a quality-by-design approach, an optimized formulation (4% AZM, 20% leucine, and 76% mannitol) was achieved. **Results:** The formulation demonstrated excellent aerodynamic properties with a mass median aerodynamic diameter (MMAD) of 2.72 μm ± 0.01 μm and fine particle fraction (FPF) (<5 μm) of 65.42% ± 5.12%. AZM-loaded microparticles exhibited enhanced efficacy against *Pseudomonas aeruginosa* with a two-fold reduction in the minimum bactericidal concentration (7.81 μg/mL vs. 15.62 μg/mL) compared to unprocessed AZM, while maintaining activity against *Streptococcus pneumoniae*. AZM microparticles demonstrated good biocompatibility with red blood cells and bronchial epithelial cells at therapeutic concentrations. **Conclusions:** These findings establish a promising lactose-free antibiotic formulation for targeted pulmonary delivery with enhanced antimicrobial efficacy.

## 1. Introduction

The lung is the most frequent site of bacterial infection, bearing in mind the great exchange area available between the environment and the internal milieu [1]. Respiratory diseases affect the upper and lower airways, nasal passages, bronchi, and lungs. Lower respiratory infections due to bacteria such as *Pseudomonas aeruginosa*, which is mainly responsible for pulmonary exacerbation in the cystic fibrosis (CF) population, and other microorganism, such as *Staphylococcus aureus*, *Streptococcus pneumoniae*, *Haemophilus influenzae* type b, influenza, respiratory syncytial virus and now also the SARS-CoV-2 virus, are a leading cause of morbidity and mortality around the world [2].

Antibiotics such as macrolides are widely used in the treatment of lung infections. The drug of choice among macrolides is azithromycin (AZM), a second-generation, broad-spectrum antibiotic effective against a broad range of bacterial and mycobacterial infections. It also possesses anti-inflammatory, immune-modulatory, and antiviral properties, which are of potential interest for treating the SARS-CoV-2 virus. Its broad antibacterial spectrum, exceptional pharmacokinetics, and immune-modulatory effects contribute to its efficacy in managing conditions such as acute bacterial sinusitis and community-acquired pneumonia. AZM’s ability to penetrate tissues extensively and sustain its presence enhances its therapeutic impact, making it a valuable option for respiratory tract infections [3,4].

However, the oral and parenteral administration of AZM results in high systemic antibiotic concentrations not only in the respiratory tract but also in the rest of the body, which can trigger toxicity, especially in immunocompromised patients. For example, oral and intravenous administration of broad-spectrum antibiotics also disrupts the normal gut flora, increases the risk for secondary infections such as *Clostridium difficile*, and may promote drug resistance [5]. Hence, inhalation would be an ideal route of administration for the treatment of bronchial infections. In clinical practice, most drugs are not designed to be administered by inhalation. Innate pulmonary defences can effectively eliminate antibiotic particles delivered via inhalation, particularly if these particles are not optimally formulated for this route [6]. Microencapsulation addresses these challenges by encapsulating AZM in biocompatible polymers, which help to protect the drug from degradation, control its release rate, and improve its aerosolization properties. This strategy has shown potential in enhancing the local delivery of AZM directly to the lungs, allowing for a targeted and efficient treatment of infections in the lower respiratory tract [7,8].

Dry powder inhalers (DPIs) are a significant advancement in inhaled drug therapy, offering a stable and efficient method for delivering medication directly to the lungs. Unlike nebulizers and pressurized metered-dose inhalers (pMDIs), DPIs deliver drugs in a dry powder form, which can be advantageous in terms of stability and ease of use. The effectiveness of DPIs is largely dependent on the formulation of the powder and the design of the inhaler device, which together ensure that the drug is adequately dispersed and delivered to the target site in the respiratory tract. DPIs are breath-actuated. Therefore, it is not necessary to synchronize inhalation with actuation, facilitating patient compliance and inhaler efficacy [9,10].

Lactose has been the most commonly used carrier in DPIs due to its excellent flow properties, stability, and compatibility with a wide range of drugs [11]. However, lactose-based DPIs have several limitations. The Maillard reaction, a chemical reaction between lactose and drugs, can lead to the formation of potentially harmful compounds [12,13]. Additionally, lactose can cause hypersensitivity reactions in some patients, and its use is contraindicated in individuals with lactose intolerance [14]. The development of lactose-free DPIs has focused on identifying alternative carriers that can mimic the flow and dispersion properties of lactose while avoiding its limitations.

Mannitol, a sugar alcohol, has been widely studied as a lactose-free carrier. It is a mucolytic agent and can enhance the aerosolization properties of DPIs. Mannitol-based DPIs have shown improved fine particle fractions and higher lung deposition compared to lactose-based DPIs [15,16,17]. Leucine, an amino acid, has been used in combination with other carriers to enhance the physicochemical and aerosolization properties of DPIs. Leucine-based DPIs have shown improved flowability and dispersion, leading to higher lung deposition [18,19,20]. Also, the co-spray drying of mannitol with leucine can enhance aerosolization performance, suggesting that mannitol–leucine combinations can serve as effective carriers in lactose-free DPI formulations [15]. These findings indicate that leucine and mannitol are promising alternatives to lactose in DPI formulations, offering improved stability, performance, and aerosolization properties while mitigating the risks associated with lactose use.

This work aims at developing a lactose-free DPI formulation containing AZM that delivers the drug directly to the site of action, improving lung deposition and controlling its release to decrease body toxicity and increase efficacy compared to other oral and intravenous treatments for acute lung infections. Hence, microencapsulation in suitable carriers could allow for an optimal lung deposition targeting an aerodynamic particle size between 1 µm and 5 µm. This work aimed to develop a DPI based on lactose-free microparticles loaded with AZM to target lung infections more efficiently. A quality-by-design approach was applied to investigate the effect of different excipients on particle size and yield. An extensive physicochemical and biological characterization was performed.

## 2. Materials and Methods

### 2.1. Materials

AZM with a purity of ≥95% was bought from Kemprotec (Cumbria, UK), D(−)-mannitol was acquired from Merck Millipore (Darmstadt, Germany), and leucine (98% purity) was bought from Sigma Aldrich (Madrid, Spain). Phosphoric acid (85%) (H_3_PO_4_) and sodium hydroxide (NaOH) were bought from Panreac S.A. (Barcelona, Spain). HPLC grade solvents were used. All other chemicals were of reagent grade and were used without further purification.

### 2.2. Methods

#### 2.2.1. Design of Experiments (DoE)

A quality-by-design approach was used to find the optimal ratio between excipients and AZM. The software Design Expert^®^ version 10.0 (M/s Stat-Ease, Minneapolis, MN, USA) was employed for factor screening. A two-factor, twenty-run design was employed to perform the screening of several process and formulation parameters. Two numerical factors affecting the development of the AZM DPI formulation were selected: (i) the amount of AZM between 1.0% and 13.1%, and (ii) the amount of leucine ranging between 10.0 and 23.4%. This design has the specific advantage of requiring a minimal number of experiments (*n* = 20), including a large number of levels per factor [21]. The resulting models were analysed to determine the main effects on yield and the percentage of particles between 1 µm and 5 µm, which were visualized using 3D surface plots to illustrate the influence of each factor.

Microparticles were prepared from an aqueous suspension (4% *w*/*v*, 1 g/250 mL) consisting of AZM (1–13.1%; *w*/*w*) and leucine (20% *w*/*w*) based on the DoE matrix (Table 1). The amount of mannitol (ranging from 70 to 90%; *w*/*w*) was adjusted to complete 100% (1 g batch size). All components were mixed previously using a magnetic stirrer for 1 h before spray drying in a Büchi B191 Mini Spray Dryer (Büchi Labortechnik AG, Flawil, Switzerland) using a high-efficiency cyclone in an open mode. The 125 °C inlet temperature, 5 mL/min solution feed rate (equivalent to 10%), 800 NL/h flow rate, and 100% aspirator force were kept constant. The suspension was stirred continuously at 300 rpm using a magnetic stirrer during spray drying to maintain homogeneity.

Once the suspension was spray-dried, the particles were collected, and the following two (2) responses were evaluated: yield and geometric particle size. The yield was calculated using Equation (1):

(1) $$Yield\left(\%\right)=\frac{Weight\;of\;collected\;powder}{Weight\;of\;solutes\;in\;the\;initial\;feed\;solution}\times 100\%$$

The particle size distribution based on geometry was analyzed through laser diffraction utilizing a Malvern^®^ Mastersizer 2000 (Malvern Instruments Ltd., Worcestershire, UK). To disperse the AZM microparticles, a Scirocco dry powder feeder was employed, operating at a pressure of 3 bar with a vibration feed setting of 75%, targeting an obscuration range between 0.5% and 3%. The findings were presented as the median particle diameter (D50) and the proportion of particles falling within the 1 µm to 5 µm size range.

*Drug loading and drug encapsulation*

High-performance liquid chromatography (HPLC) was carried out using a Varian Prostar 230 solvent delivery system, accompanied by a Varian Prostar 410 autosampler and a Varian Prostar 310 UV-visible detector (Varian^®^, Palo Alto, CA, USA). Chromatographic data were acquired and analyzed using the Galaxie Chromatography Data System (Varian^®^, CA, USA). The separation of AZM was achieved using a Thermo Scientific BDS Hypersil C18 reverse-phase column (250 mm × 4.6 mm, 5 μm particle size). A previously validated HPLC method by Al-Hakkani et al., 2019 [22]. The mobile phase consisted of phosphate buffer (0.2 M KH_2_PO_4_, pH 8) and methanol in a 1:10 volume ratio at a flow rate of 1.2 mL/min, injection volume of 50 μL, and the detector set at 210 nm. A linear calibration curve for unprocessed AZM was established within the concentration range of 50 µg/mL to 800 µg/mL, yielding a correlation coefficient of *R^2^* = 0.989 and the regression equation y = 0.0356x − 0.0154.

Drug loading (DL) and encapsulation efficiency (EE) of AZM were determined (Equations (2) and (3), respectively). For this analysis, approximately 5 mg of the powdered formulation (*n* = 3) was accurately weighed, suspended in 1 mL of the mobile phase, and analyzed using HPLC.

(2) $$DL\left(\%\right)=\frac{Weight\;of\;AZM}{Weight\;of\;powder\;formulation}\times 100$$

(3) $$EE\left(\%\right)=\frac{Total\;AZM\;encapsulated}{Total\;AZM\;content}\times 100$$

*Morphology and particle size characterization*

The average particle size in aqueous suspension (5 mg/mL concentration), along with polydispersity index and zeta potential, was assessed using a Zetasizer instrument (Malvern Instruments, Malvern, UK). Particle size and polydispersity measurements were conducted at a 90° scattering angle and maintained at a temperature of 25 °C. Prior to sample analysis, 100 nm polystyrene standards were measured to validate the system, and the results aligned with the expected particle size of the standards [23].

Imaging was performed using a Transmission Electron Microscope (TEM) (JEM 1400 plus, JEOL, Tokyo, Japan), operating with an acceleration voltage between 40 kV and 120 kV. To prepare the samples, a droplet of the aqueous dispersion (5 mg/mL) was deposited onto a grid coated with Formvar and carbon. Excess liquid was carefully removed using Whatman No. 1 filter paper. The specimens were then subjected to negative staining with a 1% *w*/*v* solution of phosphotungstic acid. Image acquisition was carried out using an AMT digital camera [24].

#### 2.2.2. Solid State Characterization

*Morphology*

The shape and structure of the spray-dried optimized microparticulate formulations were examined using Scanning Electron Microscopy (SEM) (JSM 6335F JEOL, Tokyo, Japan) with a secondary electron detector operating at 15 kV. Samples were coated with a thin layer of pure gold using a metallizer before analysis (Q150RS Metalizador QUORUM, Quorum Technologies Ltd., Lewes, UK) under vacuum conditions for 180 s [25].

*Powder X-Ray Diffraction (pRXD)*

X-ray diffraction analysis of the powder was performed using a Philips^®^X’Pert-MPD X-ray diffractometer (Malvern Panalytical^®^; Almelo, The Netherlands) with Ni-filtered Cu K radiation (1.54). The analysis was conducted at 40 kV and 40 mA. The PXRD patterns were recorded with a step scan rate of 0.05°/s, covering a range from 5° to 40° on the 2-theta scale (*n* = 3). For comparative analysis, physical mixtures containing the active pharmaceutical ingredient and excipients were prepared by grinding in an agate mortar and pestle [26].

*Fourier-Transform Infrared (FTIR) Spectroscopy*

Fourier-transform infrared (FTIR) analysis was conducted on a Nicolet Nexus 670–870 instrument (Thermofisher, Madrid, Spain). The analysis covered wavelengths from 400 cm^−1^ to 4000 cm^−1^ with a 1 nm step scanning resolution. The resulting spectral data were analyzed using Spectragryph software (version 1.2.9, Oberstdorf, Germany), and normalization of the data was performed [27].

*Differential Scanning Calorimetry (DSC) coupled with Thermogravimetric Analysis (TGA)*

Thermal behavior was analyzed using simultaneous differential scanning calorimetry and thermogravimetric analysis (DSC-TGA) with an SDT Q600 system (TA Instruments, Elstree, UK), employing nitrogen as the purge gas. Approximately 5–6 mg of each powdered sample was subjected to a standard heating protocol, increasing the temperature from 25 °C to 400 °C at a rate of 10 °C per minute. Indium was used to calibrate the calorimeter as the reference material. The reported glass transition temperatures correspond to the midpoint of the thermal transition (*n* = 3) [28].

#### 2.2.3. In Vitro Haemolysis Assay

**Figure 1:**
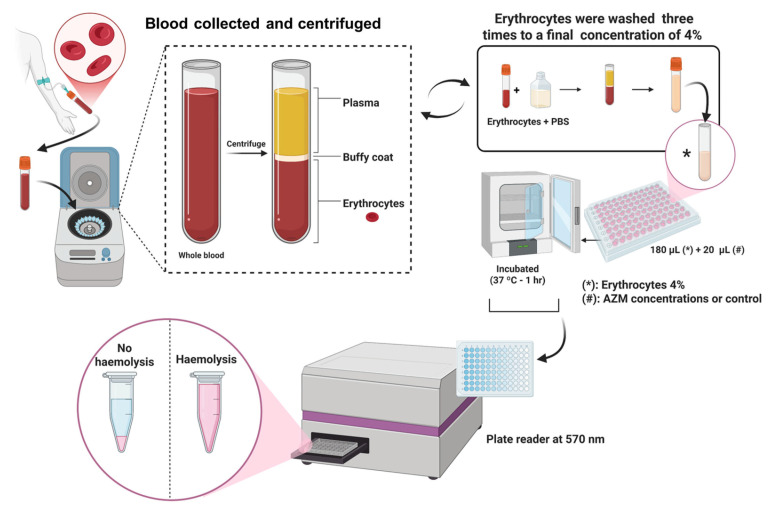
Haemolysis methodology representation.

Haemolysis studies were conducted with red blood cells (RBCs) to evaluate the formulation’s toxicity profile. Blood samples were collected from a healthy 29-year-old male volunteer in EDTA-coated Vacutainers^®^ (K2-EDTA, BD Vacutainer^®^ tubes, Becton Dickinson and Co., Franklin Lakes, NJ, USA), following ethical guidelines approved by Universidad Complutense de Madrid (Madrid, Spain). The experiment was performed as previously described [29]. Microparticles, excipients, and active pharmaceutical ingredients (APIs) were suspended in phosphate-buffered saline (PBS, 1X, pH 7.4) to prepare a series of eight serial dilutions, with AZM concentrations ranging from 200 µg/mL to 1.65 µg/mL (20 µL per well, performed in triplicate) (See Figure 1). For controls, 20% *w*/*v* Triton^®^ X-100 (Sigma-Aldrich Co., St. Louis, MO, USA) was used as the positive control, while PBS (1X, pH 7.4) served as the negative control, with both added at 20 µL per well. The samples were incubated at 37 °C for 1 h in a controlled incubator (Memmert GmBH + Co., Schwabach, Germany). After incubation, the plates were centrifuged at 1500 rpm for 5 min to pellet the intact erythrocytes. A 100 µL aliquot of the supernatant was then transferred to a transparent, flat-bottom 96-well plate. Absorbance was measured at 570 nm using a BioTek ELx808 microplate reader. Hemolysis percentage was calculated based on the corresponding Equation (4):

(4) $$\%\;Haemolysis=\frac{ABS1-ABS2}{ABS3-ABS2}\times 100$$

where ABS1 sample represents the absorbance of the sample, ABS2 is the absorbance of the negative control, and ABS3 is the absorbance of the positive control. Compusyn^TM^ v1.0 was used to calculate the concentration needed to produce 50% haemolysis (HC_50_) (Combosyn Inc., Paramus, NJ, USA).

#### 2.2.4. Antibacterial In Vitro Assay

The antimicrobial activity of the formulations was assessed against two bacterial strains, *Streptococcus pneumoniae* (NCTC 12977) and *Pseudomonas aeruginosa* (CECT 110), obtained from the Spanish Type Culture Collection (Collection Española de Cepas, Valencia, Spain). The minimum inhibitory concentration (MIC) was determined using the broth microdilution method in 96-well microplates, following the guidelines provided by the Clinical Laboratory Standards Institute (CLSI) and conducted in triplicate (*n* = 3). AZM stock solutions were initially prepared at 500 µg/mL and subjected to a series of twelve twofold (1:1 *v*/*v*) serial dilutions using Mueller-Hinton Broth (MHB) to ensure consistent AZM concentrations across all test samples.

To prepare the bacterial inoculum, cultures were separated from their growth media via centrifugation at 3000 rpm for 5 min. The supernatant was discarded, and the bacterial pellets were washed, resuspended, and diluted in 0.9% saline solution to adjust the turbidity to 0.5 McFarland standard at 600 nm. Then, 100 µL of the bacterial suspension, containing approximately 5 × 10^5^ colony-forming units (CFU/mL), was dispensed into each well and mixed with 100 µL of the corresponding AZM dilution. The plates were incubated at 37 °C for 18 to 20 h. Sterile broth was used as a negative control, while broth inoculated with bacteria but without AZM served as the positive growth control. The antimicrobial effect was evaluated by measuring the optical density at 600 nm using a Victor^TM^ X3 2030 Multilabel Reader (Perkin Elmer, Waltham, MA, USA). The MIC was identified as the lowest AZM concentration that inhibited visible bacterial growth. To determine the minimum bactericidal concentration (MBC), 100 μL from each well was transferred to Mueller–Hinton agar (MHA) plates and incubated at 37 °C for 18 h. The MBC was identified as the lowest concentration that completely inhibited bacterial colony formation.

#### 2.2.5. In Vitro Lung Deposition

Aerodynamic performance was evaluated using a Next Generation Impactor (NGI; MSP Corporation, Shoreview, MN, USA), which was connected to an HCP5 vacuum pump (Copley Scientific, Nottingham, UK) through a TPK 2000 critical flow controller (Copley Scientific, Nottingham, UK). The NGI setup included seven stainless steel collection stages, a stainless-steel induction port, and a micro-orifice collector (MOC). To minimize particle rebound and improve deposition accuracy, the collection cups were pre-coated with a 2% (*w*/*v*) solution of Tween 20 in ethanol, which was allowed to dry completely before testing. Airflow was adjusted to 60 L/min using a TSI 4000 Series Mass Flow Meter 4040 (TSI Incorporated, Shoreview, MN, USA), simulating a 4 s inhalation corresponding to a total inhaled volume of 4 L.

For aerosol performance testing, hydroxypropyl methylcellulose capsules (size No. 3) containing 25 ± 1 mg of the formulation (*n* = 3) were inserted into a Handihaler^®^ device (Boehringer, Ingelheim am Rhein, Germany). The HandiHaler^®^ was chosen due to its widespread clinical use and standardization in pulmonary drug studies. It provides consistent airflow (60 L/min) compatible with NGI measurements and simulates the inspiratory flow rate of adult patients. Additionally, it enables reproducible capsule-based dosing, which is essential during preclinical aerodynamic testing [30,31].

The deposited formulation in each NGI compartment was quantified using the previously established HPLC method. The mass median aerodynamic diameter (MMAD) and fine particle fraction (FPF, defined as particles smaller than 3 µm and 5 µm) were used to characterize in vitro deposition behavior. MMAD was calculated by plotting cumulative particle mass percentages versus aerodynamic diameters on log-probability graphs using data from all NGI stages. The FPF was determined as a percentage of the total emitted dose, calculated by comparing the mass of AZM deposited on specific impactor stages to the overall emitted amount. Stage cut-off diameters were defined according to the criteria set by Marple et al. (2003) [32].

#### 2.2.6. In Vitro Cell Culture Assays

*Cell Culture Conditions*

Human bronchial epithelial Calu-3 cells, obtained from ATCC (No. HTB-55, Lot. 61449062), were cultured in DMEM/F-12 with glutamine supplemented with 10% Fetal Bovine Serum (FBS) and 1% penicillin/streptomycin. The cells were maintained at 37 °C in a humidified incubator with 5% CO_2_.

*Cell Viability Assay*

Cell viability was assessed using the MTT assay. Calu-3 cells were plated at a density of 3.0 × 10^4^ cells per well. The cells were exposed to AZM (from 0.10 µg/mL to 50 µg/mL) for 24 h. A 5% Triton-X solution was used as a positive cytotoxic control. Following treatment, 100 µL of MTT solution (5 mg/mL) was added to each well, and the cells were incubated for 4 h in the dark. The resulting formazan crystals were solubilized using isopropyl alcohol. Absorbance measurements were taken at 550 nm using a Spectrostar BMG microplate reader (BMG LABTECH, Ortenberg, Germany). Cell viability percentages were calculated relative to untreated cells, which were considered to have 100% viability. MTT assays were performed in triplicate.

#### 2.2.7. Statistical Analysis

The statistical data analysis was conducted using a one-way ANOVA test with Minitab v.19 software (Minitab Ltd., Coventry, UK), followed by Tukey’s test at a 95% significance level. The results were visualized and graphed using Origin 2021 software (OriginLab Corporation, Northampton, MA, USA).

## 3. Results

### 3.1. DoE and Optimization of AZM Formulation

**Figure 2:**
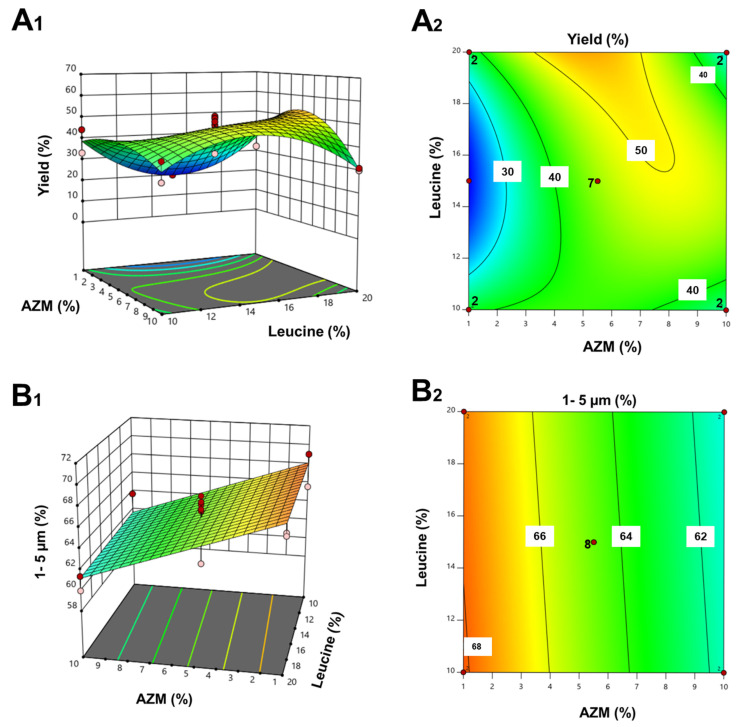
Influence of AZM and leucine on the yield and particle size. Key: (**A_1_**,**A_2_**) yield (%) and (**B_1_**,**B_2_**) particle size (µm).

The factors and responses evaluated in the DoE are shown in Table 1. Previous studies for microparticle optimization encapsulating antimicrobial drugs have shown that responses most impacted during the spray drying process were yield and particle size [33,34,35]. Yield was improved when high amounts of leucine and intermediate amounts of AZM were co-spray dried (Figure 2(A_1_,A_2_)). Regarding the geometric particle size, only the percentage of AZM played a significant role, while the amount of leucine was not significant. A linear model correlated the amount of AZM with the particle size. The lower the AZM amount, the higher the percentage of microparticles exhibiting a particle size ranging from 1 µm to 5 µm (Figure 2(B_1_,B_2_)).

Optimization studies focused on maximizing the yield as well as the percentage of microparticles within the 1–5 µm range. To balance both responses, the predicted optimal formulation consisted of 4% AZM, 20% leucine, and 76% mannitol. The optimal formulation was spray-dried using an airflow rate of 800 NL/h, a solution feed rate of 10% (equivalent to 5 mL/min), an inlet temperature of 125 °C, and an aspiration rate of 100%. Validation studies showed that both the yield and particle size were within the 95% confidence intervals. The yield obtained in the optimised formulation was 44.1% ± 5.2% with a percentage of particle size between 1 and 5 µm of 66% ± 4.8%.

### 3.2. Microparticle Characterization

Optimized AZM microparticles showed a drug loading of 2.7% ± 0.4%, and an encapsulation efficiency of 69.0% ± 3.2% (*n* = 6, with a range of 65.3% to 71.8%). After reconstitution, the average particle size was 519 nm ± 12 nm, and the particles exhibited a negative zeta potential of −20.5 mV ± 0.6 mV with a polydispersity index (PDI) of 0.38 ± 0.07, indicating good colloidal stability.

**Figure 3:**
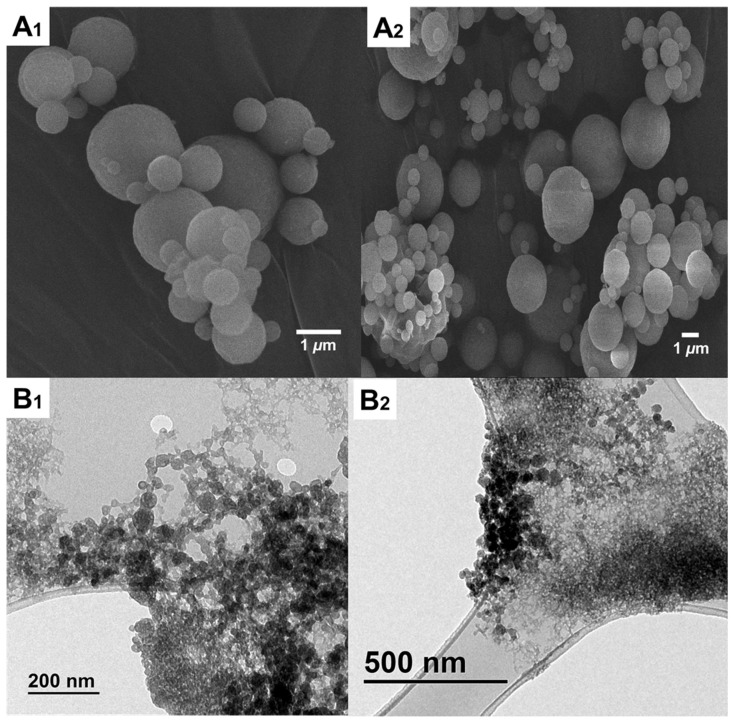
Morphological analysis. (**A_1_**,**A_2_**) SEM and (**B_1_**,**B_2_**) TEM.

The structural appearance of the AZM-loaded microparticles is presented in Figure 3. The SEM micrographs revealed spherical particles, with the majority within the range from 1 to 5 µm in size, with a smooth surface (Figure 3(A_1_,A_2_)). Particles below 1 µm were also visualised. After reconstitution in aqueous media, TEM revealed that particles were dissociated into smaller aggregates below 50 nm (Figure 3(B_1_,B_2_)).

### 3.3. Solid State Characterization

*PRXD and FT-IR analysis*

**Figure 4:**
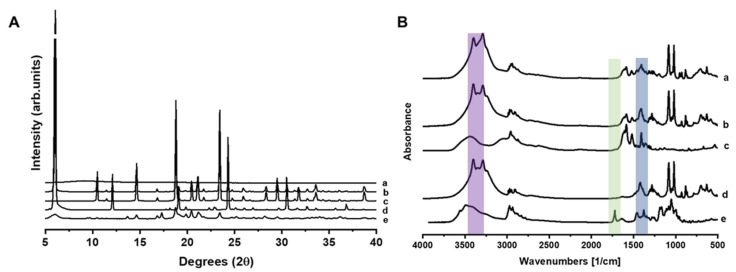
pXRD analysis (**A**) and FTIR spectra (**B**). Key: (a) AZM-MP, (b) Physical mixture, (c) Unprocessed mannitol, (d) Unprocessed leucine, and (e) Unprocessed AZM.

The pXRD analysis of unprocessed mannitol, leucine, and AZM showed Bragg peaks characteristic of crystalline structures (Figure 4A). Physical mixtures showed peaks attributed to β-mannitol, AZM, and leucine. However, no Bragg peaks were observed after spray drying, attributed to the amorphous nature of the AZM-loaded microparticles (AZM-MP). FT-IR analysis of AZM-MP revealed a shift in the N-H/O-H stretching at 3400 cm^−1^ compared to the physical mixture, which is attributed to the leucine functional groups. The observed peak shifts indicate the presence of hydrogen bonding interactions involving the amino groups of leucine, the carbonyl group of AZM, and the hydroxyl groups of mannitol (at 1420 cm^−1^). These spectral changes were more pronounced in the formulations than in the physical mixtures or raw components, strongly supporting the occurrence of intermolecular hydrogen bonding within the prepared formulations (Figure 4B). The peak in the 1750 cm^−1^ region from the AZM attributed to the cabony group was not present in the AZM-MP nor the physical mixture, probably due to a dilution effect.

*DSC-TGA analysis*

**Figure 5:**
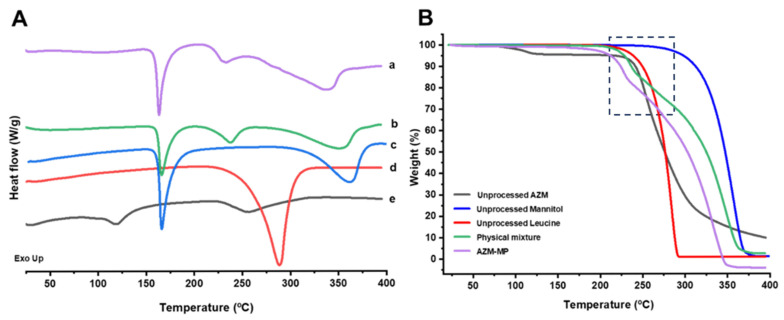
(**A**) DSC and (**B**) TGA. Keys: (a) AZM-MP (purple), (b) Physical mixture (green), (c) Unprocessed mannitol (blue), (d) Unprocessed leucine (red), and (e) Unprocessed AZM (black).

AZM dihydrate exhibited a distinct endothermic peak corresponding to dehydration at 120.4 °C ± 1.3 °C, followed by thermal decomposition, as shown in Figure 5A. This observation aligns with thermogravimetric analysis (TGA), which confirmed a significant water loss occurring at around 120 °C. In the case of unprocessed leucine, a pronounced endothermic peak was observed at 286.8 °C ± 0.2 °C, with a measured heat of fusion of 903.7 J/g ± 0.2 J/g. TGA results indicated that leucine contains no bound water and remains thermally stable up to approximately 255 °C. Similarly, unprocessed mannitol displayed a sharp melting peak at 166.2 °C ± 0.1 °C, along with its corresponding heat of fusion of 385.5 J/g ± 0.3 J/g characteristic of β-mannitol, followed by degradation above 300 °C. AZM-MP showed a similar pattern as the physical mixture. The first peak at 166 °C can be attributed to the melting of β-mannitol, while the second peak can be attributed to a depression of the melting point of leucine, followed by degradation. Bearing in mind the amorphous nature of the AZM-MP in the XRD, it is suggested that an in situ crystallization of mannitol occurs during the DSC run. The TGA pattern for the AZM-MP did not show the dehydration of AZM, as water loss probably occurred during the spray drying process. The AZM-MP exhibited a more sensitive temperature profile than the physical mixture, starting with a sharp degradation at 200 °C.

### 3.4. In Vitro Assessment of Aerodynamic Performance

**Figure 6:**
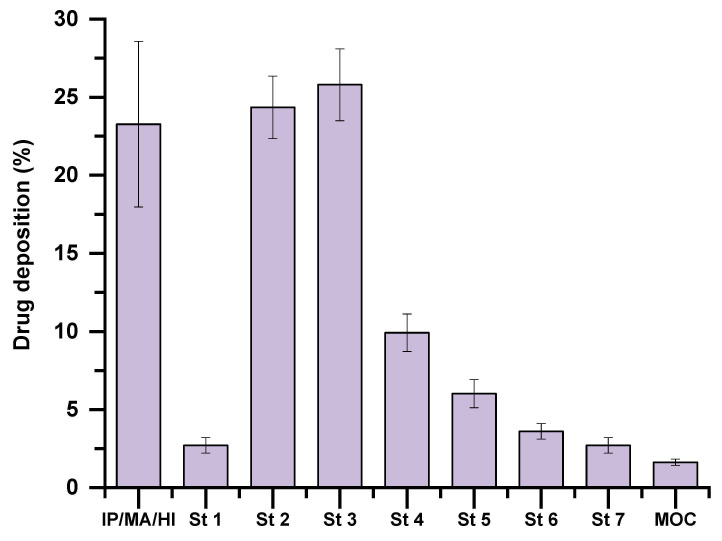
AZM deposition in different stages of the NGI. Key: (IP/MA/HI) device + mouth adaptor + induction port (St) stage, and (MOC) micro-orifice collector. Data are expressed as mean ± SD (*n* = 3).

The in vitro deposition profile of AZM-MP across the NGI stages is detailed in Figure 6. The percentage of drug recovered was as follows: induction port and mouthpiece (IP/MA/HI): 23.27% ± 5.3%; Stage 1: 2.71% ± 0.5%; Stage 2: 24.35% ± 2.0%; Stage 3: 25.79% ± 2.3%; Stage 4: 9.92% ± 1.2%; Stage 5: 6.02% ± 0.9%; Stage 6: 3.61% ± 0.5%; Stage 7: 2.71% ± 0.5%; Micro-Orifice Collector (MOC): 1.62% ± 0.2%.

The aerodynamic performance of AZM-MP demonstrated an optimal in vitro deposition with appropriate MMAD (Figure 6). The in vitro deposition profile of AZM-MP exhibited a fine particle fraction (FPF) < 5 μm of 65.42% ± 5.12% and an FPF < 3 μm of 41.59% ± 4.32%, with an MMAD of 2.72 μm ± 0.01 μm.

The emitted dose (ED), defined as the percentage of AZM recovered from the NGI stages (1–7) and the MOC, was calculated as 76.73% ± 4.4% (*n* = 3). According to standard practice, the drug deposited in the induction port is often included in ED calculations [32]. However, in our study, the induction port, mouthpiece, and device chamber were analysed as a single combined unit (IP/MA/HI), making it impossible to isolate the induction port fraction. Therefore, to avoid overestimating the ED, the entire IP/MA/HI region (23.27% ± 5.3%) was excluded from the calculation. This approach provides a conservative estimate of the emitted dose while maintaining consistency across all tested samples.

### 3.5. Antibacterial In Vitro Assay

**Figure 7:**
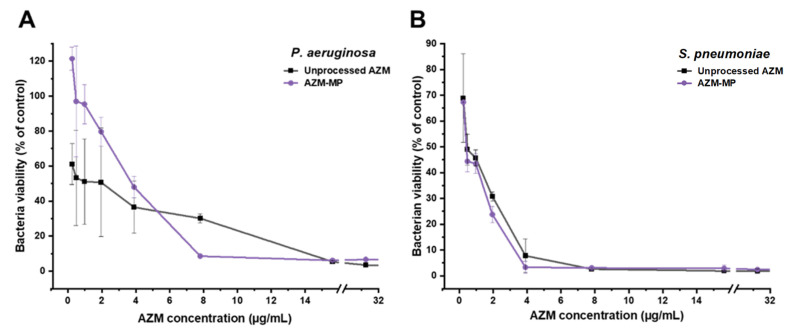
Antibacterial Efficacy at different AZM concentrations. Keys: (**A**) *P. aeruginosa* and (**B**) *S. pneumoniae*.

Both *P. aeruginosa* and *S. pneumoniae* demonstrated susceptibility to unprocessed AZM and AZM-MP formulation, though with differential response patterns (Figure 7). Against *P. aeruginosa* (Figure 7A), the AZM-MP formulation exhibited significantly enhanced antimicrobial efficacy compared to unprocessed AZM. The minimum bactericidal concentration (MBC) was determined to be 7.81 μg/mL for AZM-MP, representing a two-fold increase in potency compared to unprocessed AZM, which required 15.62 μg/mL to achieve equivalent bactericidal effects. In contrast, when tested against *S. pneumoniae* (Figure 7B), both unprocessed AZM and AZM-MP demonstrated comparable antimicrobial activity with nearly identical dose-response. The MBC was determined to be 7.81 μg/mL for both.

### 3.6. In Vitro Haemolysis and In Vitro Cytotoxicity MTT Assay

**Figure 8:**
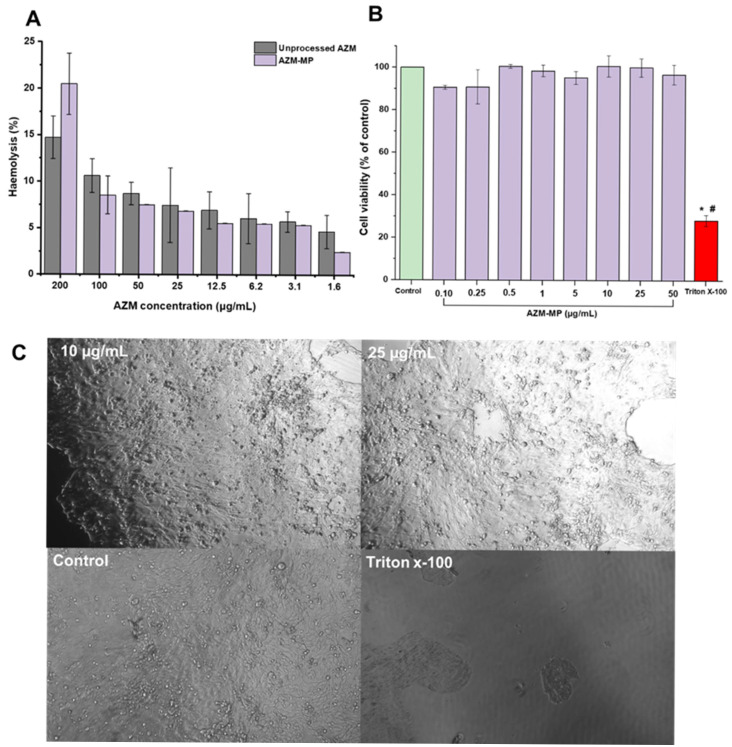
(**A**) In vitro haemolysis. (**B**) In vitro cytotoxicity. Data are expressed as mean ± SD (*n* = 5). * *p* < 0.05 vs. control and # *p* < 0.05 vs. formulation. (**C**) Cell morphology of Calu-3 cells micrographs with 10 µg/mL and 25 µg/mL AZM concentration for 24 h.

Figure 8A shows the haemolytic toxicity of AZM-MP and unprocessed AZM across different concentrations, ranging from 1.6 µg/mL to 200 µg/mL. Both demonstrated minimal haemolytic activity (<25%) at concentrations up to 200 μg/mL, with haemolysis decreasing proportionally at lower concentrations. Microencapsulation did not significantly alter the haemocompatibility profile of AZM, supporting the safety of the formulation for pulmonary administration.

Figure 8B shows the cell viability of Calu-3 cells after 24 h exposure to the AZM-MP formulation at concentrations ranging from 0.1 to 50 µg/mL. No significant differences in cell viability were observed between the AZM-MP formulation and untreated cells. Cell viability remained above 90% across all AZM concentrations. Additionally, no morphological changes were observed in cells treated with the AZM-MP formulation (Figure 8C).

## 4. Discussion

The development of lactose-free DPI loaded with AZM represents a significant advancement in pulmonary drug delivery systems for respiratory infections. This study successfully demonstrates that mannitol–leucine combinations can effectively replace lactose as carrier excipients while maintaining excellent aerodynamic performance. When comparing our formulation with electrospray excipient-free azithromycin microparticles reported by Arauzo et al. (2021) [36], several parallel outcomes emerge despite the different technological approaches. Both systems achieved particles within the optimal respiratory deposition range (MMAD 2.72 μm vs. 2.5 μm) and demonstrated excellent biocompatibility with Calu-3 bronchial epithelial cells. Both studies confirmed antimicrobial efficacy against *P. aeruginosa*, while our formulation showed enhanced potency with a two-fold reduction in MBC values. Our haemolysis assays further validated the safety profile, showing minimal erythrocyte damage even at concentrations up to 200 μg/mL, well above the therapeutic range.

Polymers for the microencapsulation of AZM. This demonstrates that carbohydrate-based microencapsulation of AZM not only improved the stability of the drug but also enhanced its aerosolization properties, which are crucial for efficient lung deposition when delivered via DPIs. The study revealed that these formulations could provide better control over the particle size and release profile, leading to a more sustained therapeutic effect in the lungs [8,35].

Our approach shares similarities with co-engineered macrolide-mannitol particles designed to treat bronchiectasis using AZM and mannitol [37]. These formulations exhibited promising aerosol performance when delivered via a novel high-payload Orbital inhaler device. Specifically, the co-spray dried formulation containing 50:50 *w*/*w* AZM achieved 57.6% ± 7.6% delivery efficiency with an FPF (<6.8 μm) of 80.4% ± 1.1% and minimal throat deposition (5.3% ± 0.9%). Our optimized formulation (4% AZM, 20% leucine, and 76% mannitol) represents a further refinement of this concept. The strategic inclusion of leucine as a surface-active amino acid enhances powder dispersibility and stability, as evidenced by the excellent aerodynamic properties achieved in our study: FPF (<5 μm) of 65.42% and MMAD of 2.72 μm. These results align with the established role of leucine in improving the aerosolization properties of spray-dried powders for inhalation. The beneficial effect of leucine in mannitol-based DPI formulations has been well-documented in the literature. Yang et al. (2014) [38], demonstrated that a zanamivir/mannitol/leucine formulation (1/1/3 ratio) achieved an FPF of 41.40% ± 1.1%, significantly higher than formulations without leucine (5.43% ± 1.7%). This substantial improvement highlights the critical role of leucine as a dispersibility enhancer in DPI formulations, a principle we successfully applied in our AZM-MP development.

In a related approach, mannitol carrier and drug-carrier composite particles produced via low-temperature supercritical assisted atomization (LTSAA) with L-leucine showed enhanced aerosolization properties [39]. The addition of leucine (9.1 mass %) created corrugated particles with reduced interparticle cohesion and improved flowability. When applied to beclomethasone dipropionate, this technique increased FPF by 3.4-fold (49.9% vs. 14.7%) and accelerated dissolution by 15.6 times compared to the unprocessed drug. While our study utilized spray drying rather than LTSAA, both approaches demonstrate how the strategic combination of mannitol and leucine can significantly enhance the aerosolization performance and dissolution characteristics of poorly water-soluble drugs for pulmonary delivery.

On the other hand, the acidic microenvironment created by leucine dissolution can enhance the dissolution rate of poorly water-soluble drugs such as AZM, making the formulation more effective for inhalation therapy [40]. This dissolution-enhancing effect likely contributes to the improved antimicrobial efficacy observed against *P. aeruginosa*, as the increased local concentration of solubilized AZM can better penetrate bacterial biofilms and cell membranes. The synergistic effect of leucine’s dispersibility-enhancing properties and its ability to create favourable dissolution conditions for AZM represents a significant advantage of our formulation design.

The combination of mannitol and leucine can be engineered to produce particles with optimal morphology and surface properties, leading to better aerosolization performance and lung deposition. This synergistic excipient combination allows for precise control over critical particle characteristics such as size, shape, and surface roughness. Mannitol provides structural integrity and suitable crystallinity, while leucine tends to migrate to the particle surface during spray drying, creating a shell-like structure that reduces interparticle cohesive forces and moisture uptake [41,42]. The resultant particles exhibit improved flow properties and dispersion behavior, as demonstrated by the excellent aerodynamic performance of our AZM-MP formulation. This optimization of particle engineering principles enables more efficient deep lung delivery of AZM for targeted antimicrobial activity at the infection site. The NGI further substantiates this, with the majority of AZM-MP depositing in stages 2 to 4, corresponding to aerodynamic diameters of approximately 2.1 µm–4.7 µm. This aerodynamic range is ideal for deposition in the small conducting airways and terminal bronchioles, where pathogens such as *P. aeruginosa* and *S. pneumoniae* commonly colonize in diseases such as cystic fibrosis and Chronic obstructive pulmonary disease (COPD) [43,44,45]. The calculated ED of about 76.73% supports the effective aerosolization and lung delivery potential of the formulation.

For clinical application, the relationship between epithelial lining fluid (ELF) volume and drug concentration is crucial for ensuring therapeutic efficacy. ELF constitutes approximately 0.39% of total lung capacity, with a volume of about 23.4 mL in healthy adults [46,47]. Based on our MBC values (7.81 μg/mL against *P. aeruginosa* and *S. pneumoniae*), we targeted a well-above concentration (approximately 40 μg/mL in ELF), providing a safety margin above the minimum inhibitory concentration while remaining well below concentrations showing any haemolytic or cytotoxic effects. Considering our drug loading of 2.7% and FPF of 65.42%, the inhalation of 25 mg of our formulation would deliver approximately 437 μg of AZM to the lungs. Assuming an average ELF volume of 20 mL, this would result in an estimated concentration of 21.9 μg/mL. The inhalation of two capsules would result in effective concentrations for *P. aeruginosa* and *S. pneumoniae*. The favourable safety profile of our formulation, with >90% cell viability at concentrations up to 50 μg/mL, supports the feasibility of this dosing approach for clinical application.

Beyond aerodynamic deposition, the formulation’s fine particle size and surface-modified morphology are likely to influence its interaction with the lung’s mucosal and cellular environment. The substantial FPF < 3 µm (41.59%) suggests that a significant portion of the dose may reach alveolar and terminal airway regions, where clearance mechanisms differ from ciliated airways. In these zones, reduced mucociliary clearance and lower immune surveillance may enhance drug residence time, potentially extending the local therapeutic window [48,49]. Moreover, the moderate zeta potential (–20.5 mV) implies adequate colloidal stability while maintaining surface characteristics that favor epithelial uptake without triggering aggregation. These attributes, combined with the observed reduction in MBC, support the hypothesis that particle design in AZM-MP not only improves delivery but may also potentially develop localized drug activity at the infection site.

## 5. Conclusions

The development of lactose-free dry powder inhalers loaded with AZM represents a significant advancement in pulmonary drug delivery systems for respiratory infections. This study successfully demonstrates that mannitol–leucine combinations can effectively replace lactose as carrier excipients while maintaining excellent aerodynamic performance. The microencapsulated AZM exhibited enhanced antimicrobial efficacy against bacterial pathogens such as *P. aeruginosa*, while maintaining a favorable safety profile. These findings provide a promising alternative for patients with lactose intolerance and offer potential therapeutic advantages in targeting resistant pulmonary infections.

## Figures and Tables

**Table 1:** DoE matrix including dependent and independent factors.

Experiment	AZM (%)	Leucine (%)	Yield (%)	Percentage of Particle Size in the Range Between 1 and 5 µm (%)
1	1.0	10.0	33.0	65.6
2	10.0	10.0	30.0	58.1
3	1.0	20.0	32.3	66.5
4	10.0	20.0	31.0	59.9
5	5.5	15.0	-	59.9
6	1.0	15.0	20.0	-
7	13.1	15.0	33.2	58.5
8	5.5	6.6	40.7	67.4
9	5.5	23.4	59.8	66.6
10	5.5	15.0	44.8	66.1
11	5.5	15.0	48.0	65.3
12	5.5	15.0	52.0	65.9
13	5.5	15.0	51.0	66.8
14	5.5	15.0	49.5	66.2
15	5.5	15.0	42.0	65.5
16	1.0	10.0	44.0	69.1
17	10.0	10.0	38.5	63.7
18	1.0	20.0	41.7	66.8
19	10.0	20.0	30.0	61.4
20	5.5	15.0	35.0	63.9

## Data Availability

Data will be made available upon request.

## References

[1] Kradin R.L., Digumarthy S. (2017). The pathology of pulmonary bacterial infection. Semin. Diagn. Pathol..

[2] Troeger C., Blacker B., Khalil I.A., Rao P.C., Cao J., Zimsen S.R., Albertson S.B., Deshpande A., Farag T., Abebe Z. (2018). Estimates of the global, regional, and national morbidity, mortality, and aetiologies of lower respiratory infections in 195 countries, 1990–2016: A systematic analysis for the Global Burden of Disease Study 2016. Lancet Infect. Dis..

[3] Firth A., Prathapan P. (2021). Broad-spectrum therapeutics: A new antimicrobial class. Curr. Res. Pharmacol. Drug Discov..

[4] Parnham M.J., Haber V.E., Giamarellos-Bourboulis E.J., Perletti G., Verleden G.M., Vos R. (2014). Azithromycin: Mechanisms of action and their relevance for clinical applications. Pharmacol. Ther..

[5] Burke D., Harrison M., Fleming C., McCarthy M., Shortt C., Sulaiman I., Murphy D., Eustace J., Shanahan F., Hill C. (2017). *Clostridium difficile* carriage in adult cystic fibrosis (CF); implications for patients with CF and the potential for transmission of nosocomial infection. J. Cyst. Fibros..

[6] Wenzler E., Fraidenburg D.R., Scardina T., Danziger L.H. (2016). Inhaled antibiotics for gram-negative respiratory infections. Clin. Microbiol. Rev..

[7] Chan S.H.Y., Sheikh K., Zariwala M.G., Somavarapu S. (2023). Dry powder formulation of azithromycin for COVID-19 therapeutics. J. Microencapsul..

[8] Anaya B.J., Kara A., Raposo R., Tirado D.F., Lalatsa A., González-Burgos E., Serrano D.R. (2025). Integration of 3D-printed micromixers and spray drying for pulmonary delivery of antimicrobial microparticles. Int. J. Pharm..

[9] Cheng Y.S. (2014). Mechanisms of pharmaceutical aerosol deposition in the respiratory tract. AAPS PharmSciTech.

[10] Khan I., Elhissi A., Shah M., Alhnan M.A., Ahmed W. (2013). Liposome-based carrier systems and devices used for pulmonary drug delivery. Biomaterials and Medical Tribology.

[11] Hebbink G.A., Jaspers M., Peters H.J., Dickhoff B.H. (2022). Recent developments in lactose blend formulations for carrier-based dry powder inhalation. Adv. Drug Deliv. Rev..

[12] Gholizadeh-Hashjin A., Hamishehkar H., Monajjemzadeh F. (2024). Detection of Excipient–Excipient Interaction in Dry Powder Inhaler Formulation Prepared by Spray Drying. Pharm. Sci..

[13] Agnihotri V.V., Gorle A.P. (2024). Quality by design based synthesis and characterization of novel maleyl functionalized albumin solid dry powder for pulmonary targeting. Dry. Technol..

[14] Bar-On O., Levine H., Stafler P., Shmueli E., Jacobi E., Goldberg O., Steuer G., Prais D., Mei-Zahav M. (2022). Lactose-Containing Dry-Powder Inhalers for Patients with Cow’s Milk Protein Allergy—The Conundrum; A National Survey of Pediatric Pulmonologists and Allergologists. J. Clin. Med..

[15] Molina C., Kaialy W., Nokhodchi A. (2019). The crucial role of leucine concentration on spray dried mannitol-leucine as a single carrier to enhance the aerosolization performance of albuterol sulfate. J. Drug Deliv. Sci. Technol..

[16] Muralidharan P., Mallory E.K., Malapit M., Phan H., Ledford J.G., Hayes D., Mansour H.M. (2020). Advanced design and development of nanoparticle/microparticle dual-drug combination lactose carrier-free dry powder inhalation aerosols. RSC Adv..

[17] Banat H., Csóka I., Paróczai D., Burian K., Farkas Á., Ambrus R. (2024). A Novel Combined Dry Powder Inhaler Comprising Nanosized Ketoprofen-Embedded Mannitol-Coated Microparticles for Pulmonary Inflammations: Development, In Vitro–In Silico Characterization, and Cell Line Evaluation. Pharmaceuticals.

[18] Ordoubadi M., Shepard K.B., Wang H., Wang Z., Pluntze A.M., Churchman J.P., Vehring R. (2023). On the physical stability of leucine-containing spray-dried powders for respiratory drug delivery. Pharmaceutics.

[19] Ferdynand M.S., Nokhodchi A. (2020). Co-spraying of carriers (mannitol-lactose) as a method to improve aerosolization performance of salbutamol sulfate dry powder inhaler. Drug Deliv. Transl. Res..

[20] Lamy B., Serrano D.R., O’connell P., Couet W., Marchand S., Healy A.M., Tewes F. (2019). Use of leucine to improve aerodynamic properties of ciprofloxacin-loaded maltose microparticles for inhalation. Eur. J. Pharm. Res..

[21] Shukla A., Mishra V., Bhoop B.S., Katare O.P. (2015). Alginate coated chitosan microparticles mediated oral delivery of diphtheria toxoid (Part A). Systematic optimization, development and characterization. Int. J. Pharm..

[22] Al-Hakkani M.F. (2019). A rapid, developed and validated RP-HPLC method for determination of azithromycin. SN Appl. Sci..

[23] Serrano D.R., Lalatsa A., Dea-Ayuela M.A., Bilbao-Ramos P.E., Garrett N.L., Moger J., Guarro J., Capilla J., Ballesteros M.P., Schatzlein A.G. (2015). Oral particle uptake and organ targeting drives the activity of amphotericin B nanoparticles. Mol. Pharm..

[24] Smith L., Serrano D.R., Mauger M., Bolás-Fernández F., Dea-Ayuela M.A., Lalatsa A. (2018). Orally bioavailable and effective buparvaquone lipid-based nanomedicines for visceral leishmaniasis. Mol. Pharm..

[25] Rapti C., Luciano F.C., Anaya B.J., Ramirez B.I., Ongoren B., Dea-Ayuela M.A., Lalatsa A., Serrano D.R. (2024). Amphotericin B Ocular Films for Fungal Keratitis and a Novel 3D-Printed Microfluidic Ocular Lens Infection Model. J. Fungi.

[26] Anaya B.J., Cerda J.R., D’Atri R.M., Yuste I., Luciano F.C., Kara A., Ruiz H.K., Ballesteros M.P., Serrano D.R. (2023). Engineering of 3D printed personalized polypills for the treatment of the metabolic syndrome. Int. J. Pharm..

[27] Anaya B.J., Raudone L., Ureña-Vacas I., Sanz-Perez A., Marksa M., Vilkickyte G., García-Rodríguez J.J., Serrano D.R., González-Burgos E. (2025). Origanum vulgare ssp. hirtum: From Plant to 3D-Printed Gummies with Antioxidant and Anti-Inflammatory Properties. Gels.

[28] Santamaría K.J., Anaya B.J., Lalatsa A., González-Barranco P., Cantú-Cárdenas L., Serrano D.R. (2024). Engineering 3D printed gummies loaded with metformin for paediatric use. Gels.

[29] Pineros I., Slowing K., Serrano D.R., de Pablo E., Ballesteros M.P. (2017). Analgesic and anti-inflammatory controlled-released injectable microemulsion: Pseudo-ternary phase diagrams, in vitro, ex vivo and in vivo evaluation. Eur. J. Pharm. Sci..

[30] Altman P., Wehbe L., Dederichs J., Guerin T., Ament B., Moronta M.C., Pino A.V., Goyal P. (2018). Comparison of peak inspiratory flow rate via the Breezhaler^®^, Ellipta^®^ and HandiHaler^®^ dry powder inhalers in patients with moderate to very severe COPD: A randomized cross-over trial. BMC Pulm. Med..

[31] Van Noord J., Cornelissen P., Aumann J.-L., Platz J., Mueller A., Fogarty C. (2009). The efficacy of tiotropium administered via Respimat^®^ Soft Mist^TM^ Inhaler or HandiHaler^®^ in COPD patients. Respir. Med..

[32] Marple V.A., Roberts D.L., Romay F.J., Miller N.C., Truman K.G., Van Oort M., Olsson B., Holroyd M.J., Mitchell J.P., Hochrainer D. (2003). Next generation pharmaceutical impactor (a new impactor for pharmaceutical inhaler testing). Part I: Design. J. Aerosol Med..

[33] de Pablo E., O’Connell P., Fernández-García R., Marchand S., Chauzy A., Tewes F., Dea-Ayuela M.A., Kumar D., Bolás F., Ballesteros M. (2023). Targeting lung macrophages for fungal and parasitic pulmonary infections with innovative amphotericin B dry powder inhalers. Int. J. Pharm..

[34] Celi S.S., Fernández-García R., Afonso-Urich A.I., Ballesteros M.P., Healy A.M., Serrano D.R. (2023). Co-delivery of a high dose of amphotericin B and Itraconazole by means of a dry powder inhaler formulation for the treatment of severe fungal pulmonary infections. Pharmaceutics.

[35] Anaya B.J., D’Angelo D., Bettini R., Molina G., Sanz-Perez A., Dea-Ayuela M.A., Galiana C., Rodríguez C., Tirado D.F., Lalatsa A. (2025). Heparin-azithromycin microparticles show anti-inflammatory effects and inhibit SARS-CoV-2 and bacterial pathogens associated to lung infections. Carbohydr. Polym..

[36] Arauzo B., Lopez-Mendez T.B., Lobera M.P., Calzada-Funes J., Pedraz J.L., Santamaria J. (2021). Excipient-free inhalable microparticles of azithromycin produced by electrospray: A novel approach to direct pulmonary delivery of antibiotics. Pharmaceutics.

[37] Young P.M., Salama R.O., Zhu B., Phillips G., Crapper J., Chan H.-K., Traini D. (2015). Multi-breath dry powder inhaler for delivery of cohesive powders in the treatment of bronchiectasis. Drug Dev. Ind. Pharm..

[38] Yang Y., Yang Z., Ren Y., Mei X. (2014). Effects of formulation and operating variables on zanamivir dry powder inhalation characteristics and aerosolization performance. Drug Deliv..

[39] Wu H.-T., Li T.-H., Tsai H.-M., Chien L.-J., Chuang Y.-H. (2021). Formulation of inhalable beclomethasone dipropionate-mannitol composite particles through low-temperature supercritical assisted atomization. J. Supercrit. Fluids.

[40] Mangal S., Nie H., Xu R., Guo R., Cavallaro A., Zemlyanov D., Zhou Q. (2018). Physico-chemical properties, aerosolization and dissolution of co-spray dried azithromycin particles with l-leucine for inhalation. Pharm. Res..

[41] Peng T., Zhang X., Huang Y., Zhao Z., Liao Q., Xu J., Huang Z., Zhang J., Wu C.-Y., Pan X. (2017). Nanoporous mannitol carrier prepared by non-organic solvent spray drying technique to enhance the aerosolization performance for dry powder inhalation. Sci. Rep..

[42] Wu H.-T., Su Y.-C., Wang Y.-M., Tsai H.-M. (2018). Characterization and aerosolization performance of mannitol particles produced using supercritical assisted atomization. Chem. Eng. Res. Des..

[43] Darquenne C. (2012). Aerosol deposition in health and disease. J. Aerosol Med. Pulm. Drug Deliv..

[44] Welp A.L., Bomberger J.M. (2020). Bacterial community interactions during chronic respiratory disease. Front. Cell. Infect. Microbiol..

[45] D’Anna S.E., Maniscalco M., Cappello F., Carone M., Motta A., Balbi B., Ricciardolo F.L., Caramori G., Di Stefano A. (2021). Bacterial and viral infections and related inflammatory responses in chronic obstructive pulmonary disease. Ann. Med..

[46] Motos A., Kuti J.L., Li Bassi G., Torres A., Nicolau D.P. (2019). Is one sample enough? β-lactam target attainment and penetration into epithelial lining fluid based on multiple bronchoalveolar lavage sampling time points in a swine pneumonia model. Antimicrob. Agents Chemother..

[47] Asempa T.E., Motos A., Abdelraouf K., Bissantz C., Zampaloni C., Nicolau D.P. (2019). Efficacy of human-simulated epithelial lining fluid exposure of meropenem-nacubactam combination against class A serine β-lactamase-producing Enterobacteriaceae in the neutropenic murine lung infection model. Antimicrob. Agents Chemother..

[48] Adivitiya, Kaushik M.S., Chakraborty S., Veleri S., Kateriya S. (2021). Mucociliary respiratory epithelium integrity in molecular defense and susceptibility to pulmonary viral infections. Biology.

[49] Plaunt A.J., Nguyen T.L., Corboz M.R., Malinin V.S., Cipolla D.C. (2022). Strategies to overcome biological barriers associated with pulmonary drug delivery. Pharmaceutics.

